# Examining the Outcomes of Hybrid Coronary Revascularization in Acute STEMI Patients from 2015 to 2022

**DOI:** 10.1155/2024/8861704

**Published:** 2024-02-08

**Authors:** Mozhgan Bahramian, Seyed Ali Moezi bady, Maryam Bahramian, Ahmad Amouzeshi

**Affiliations:** ^1^Student Research Committee, Birjand University of Medical Sciences, Birjand, Iran; ^2^Cardiovascular Diseases Research Center, Birjand University of Medical Sciences, Birjand, Iran

## Abstract

**Background:**

The global rise of chronic diseases, especially cardiovascular disease (CVD), poses a significant public health challenge, being a leading cause of death and disability worldwide. In Iran, the surge in CVD incidence and its risk factors, along with a decrease in the age of onset, has notably increased the reliance on coronary artery bypass grafting (CABG) as a life-saving intervention. Staged hybrid coronary revascularization (HCR), which combines percutaneous coronary intervention with delayed CABG, offers a novel approach for patients with complex coronary artery disease, potentially improving survival and reducing complications. Considering the newness of this treatment method and the limitations of previous studies, we investigated the results of staged HCR in acute ST-elevation myocardial infarction (STEMI) patients in this study.

**Methods:**

This observational study was performed on consecutive patients with acute STEMI who underwent staged HCR and were referred to Valiasr and Razi hospitals in Birjand from 2015 to 2022. The required information (demographic information, angiography result, and operation side effects) was collected in a checklist. If necessary, the patients were contacted by phone. After collecting the data, they were entered into SPSS version 16 software.

**Results:**

This study was conducted on 33 patients with a mean age of 64.88 ± 9.24 years (69.7% male). The average hospital stay was 11.6 ± 8.9 days (3 to 72 days). The mean ejection fraction and syntax score were 36.5% ± 10.2% and 31.21 ± 6.7, respectively. Following surgery and during hospitalization, arrhythmias were observed, including 33.3% with premature ventricular contractions, 18.1% with atrial fibrillation, and 3.1% with ventricular tachycardia. The average number of pack cells (red blood cells that have been separated for blood transfusion) and creatinine changes before and after hybrid surgery were 640.9 ± 670.9 cc and 0.055 ± 0.07. In the follow-up, 9.09% of patients had late mortality, 6.1% of patients had urinary tract infections during hospitalization, 6.1% of patients had surgical site infections, 3.1% needed dialysis, and none of the studied patients had premature death or need for reintervention.

**Conclusions:**

The results of our study indicated that staged HCR performed early after an ACS is not associated with significant mortality or complications. Therefore, it is advisable to consider staged HCR as a surgical option in appropriate cases.

## 1. Introduction

Chronic diseases, particularly cardiovascular disease (CVD), cancer, respiratory disease, and diabetes, have emerged as leading public health challenges globally [1, 2], significantly impacting mortality rates in both developed and developing nations [3]. In the early 21^st^ century, CVD alone accounts for approximately 50% of deaths in developed countries and 25% in developing ones [4]. Based on predictions, CVD could cause up to 25 million deaths annually, with a projected increase in incidence by 20–45% in Iran [5, 6]. Various studies have stated that in Iran, the prevalence of CVD and its risk factors is growing, and the age of developing CVD is decreasing [7]. Indeed, CVD has been claimed as the first cause of mortality in Iran (300 deaths per day) [8, 9].

CVD can be caused by hereditary or acquired cardiac anomalies and significantly degrades patients' functional capacity and quality of life [7, 8]. This disease is often progressive and compromises the ability to supply sufficient blood flow to fulfill the metabolic demands of the body's organs and tissues. In these patients, reduction in exercise capacity and shortness of breath occur [9]. In addition to healthcare and economic costs, CVD is also regarded as one of the most important causes of disability and incapacitation [1]. Given the increasing prevalence of CVD, the significance of open-heart surgery, particularly coronary artery bypass grafting (CABG), has grown substantially, as it is associated with reduced mortality [10]. The majority of open-heart procedures performed in Iran involve CABG [11]. A study has reported that millions of open-heart surgeries are annually performed on patients worldwide [12].

Patients with comorbidities, inappropriate graft conduits, and complicated coronary anatomy provide a considerable therapeutic challenge in instances of acute coronary syndrome (ACS). Staged hybrid coronary revascularization (HCR) is a potential option for individuals in these circumstances. It involves performing a percutaneous coronary intervention (PCI) with a drug-eluting stent (DES) on a non-left anterior descending (LAD) lesion, followed by a delayed CABG around 3–7 days in this center [13, 14]. This method has showed that grafting the left internal mammary artery (LIMA) to the LAD yields beneficial outcomes, promoting extended survival and reducing coronary relapses, albeit with an increased risk of cerebrovascular accidents (CVAs) or stroke [15]. This therapeutic method is more suitable than CABG because of its extended openness and being free of atherosclerosis [14].

Staged HCR is a promising and new therapy method that has reduced short- and mid-term mortality and complication rates. Considering its recent debut and the lack of thorough studies testing its effectiveness, this study objectively evaluates staged HCR operations in acute ST-elevation myocardial infarction (STEMI) patients. This unique approach's therapeutic advantages and risks will be examined to enhance cardiac care literature.

## 2. Method

This observational study was performed on all patients with acute STEMI undergoing staged HCR, referring to Valiasr and Razi hospitals in Birjand from 2015 to 2022. The inclusion criteria were diagnosed STEMI and undergoing intervention, followed by delayed CABG (between three and seven days). The exclusion criteria included the impossibility of intervention or CABG, NSTEMI, elective patients, and intervention complications.

### 2.1. Sample Size and the Sampling Method

A total of 33 patients with acute STEMI underwent staged HCR at the Valiasr and Razi hospitals in Birjand between March 20, 2015, and November 21, 2022. This study was designed and implemented after approval by the Research and Ethics Committee of the Birjand University of Medical Sciences.

For data collection of patients, a checklist designed based on the study objectives was used. The study focused on patients initially referred to the emergency ward, diagnosed with severe coronary artery involvement (severe CAD) by a cardiologist. Following this, they were directed to an intervention fellowship for primary PCI. In cases where total revascularization was unattainable through PCI, we conducted primary PCI for the acute STEMI in the culprit lesion and subsequently referred patients for delayed CABG after 3–7 days of stabilization. If the patient's syntax score during primary angiography exceeded 22, and total revascularization was deemed preferable, we opted for the hybrid procedure, involving a heart team decision-making process. The CABG procedure was conducted on the pump. All patients underwent invasive routine angiography in the setting of primary PCI, and after fixing the culprit lesion, delayed CABG was performed afterward as a hybrid procedure several days later.

The executive of the study systematically extracted patient data from medical records coded 247 in the HIS system. The data extraction process was tailored to individuals who had undergone CABG. When dubious decisions were made regarding the selection of patient files, consultations with the attending surgeon or an interventional fellow were pursued. Follow-up assessments encompassed early mortality, determined through telephonic outreach, as well as morbidity incidents, including cerebral vascular accidents (CVA), surgical site infections (SSI), and sepsis, alongside urinary tract infections (UTI), elevated creatinine levels, dialysis requirements, and the need for packed red blood cell transfusions. Additionally, the prevalence and types of arrhythmias, atrial fibrillation (AF), ventricular fibrillation (VF), ventricular tachycardia (VT), and premature ventricular contractions (PVC), as well as the rate of reinterventions were evaluated from patient files. When necessary, direct phone contact with patients was established to gather comprehensive data.

### 2.2. Data Analysis Method

Upon collection, data were entered into SPSS version 16 for analysis. Descriptive statistics were summarized by using means, standard deviations, and relative frequencies. Statistical analyses were conducted with a predetermined significance threshold of 5%.

### 2.3. Ethical Considerations

The ethics code was received from the Ethics Committee of the Birjand University of Medical Sciences under the number of IR.BUMS.REC.1401.025. All of the data were analyzed and reported collectively. The data were collected while keeping the confidentiality of patient information and analyzed independently of the patient's identity.

## 3. Results

Based on the results of this study, presented in Table 1, this study was performed on 33 patients with acute myocardial infarction who underwent staged HCR. The mean interval between PCI and CABG was 4.6 days (3–7 days). The mean age of the patient was 64.88 ± 9.24 years, with the minimum and maximum ages of 44 and 83 years, respectively. The mean body mass index(BMI) of patients, ejection fraction before the hybrid surgery, hospitalization day, and syntax score before the hybrid surgery, respectively, were 24.99 ± 4.38, 36.5 ± 10.2, 8.9 ± 11.6 days (3–72 days), and 31.21 ± 6.7.

Based on the results of the present study provided in Table 2, 23 patients (69.7%) were male and 10 (30.3%) were female. Also, 36.4% of the study patients had a history of diabetes, 30.3% of them used drugs and insulin, and the others just used diet, 51.5% had a history of hypertension (HTN), and they used medication for HTN, 21.2% had a history of dyslipidemia and all of them used drug, 6.1% had a history of heart failure, and 3.1% had a history of CVA before angiography. 9.09% of the patients had a family history of CAD, and just one of the patients had previous CAD (3 years before staged HCR had anterior MI).

Based on the present study results provided in Table 3 regarding the number of involved vessels in angiography, it is observed that most patients had 3VD (three-vessel disease). The most common target vessel of angioplasty in patients was RCA. The most common type of MI in patients was inferior MI. Most patients had presurgery syntax scores between 22 and 33. The type of implanted stent in the studied patients was a coronary drug-eluting stent, which was used in 12.1% of patients, and for the rest of the patients, balloon angioplasty was performed.

Based on the present study results in Table 4, 87.9% of the study patients needed to receive packed red blood cells following the hybrid surgery. The main reception of packed red blood cells in the patient was 670.9 ± 640.9.

Based on the present study results outlined in Table 5, the mean preoperative and postoperative creatinine levels were 1.109 ± 0.55 and 1.164 ± 0.47, where the mean changes of creatinine elevation in the study patients were 0.055 ± 0.07.

Based on the present study results reported in Table 6, in the follow-up of patients, the mean follow-up was three years and two months (1–7 years). Up to now, no case of reintervention for the study patients has been observed, and 54.5% of the patients had arrhythmia following the hybrid surgery, with PVC being the most common type, followed by AF.

Based on the present study results provided in Table 7, other complications were also observed in the follow-up; none of the study patients had CVA or sepsis after the hybrid surgery; 6.1% developed UTI during hospitalization, and 3.1% needed dialysis during their hospital stay.

Based on the present study results provided in Table 8, showing the mortality outcomes of patients, it is observed that none of the patients had early mortality (mortality earlier than one month). Up to now, in the follow-up, three patients have died. The history of the patients who died is as follows:

1. A 61-year-old woman with a history of dyslipidemia, diabetes, hypertension, heart failure, and chronic kidney disease with a diagnosis of inferior MI, died 1.5 months following the hybrid surgery. The cause of mortality was septic shock.
2. A 66-year-old woman with a history of hypertension, heart failure, and CVA (eight minutes before angiography), with a diagnosis of anterior MI, died one year later because of pulmonary edema and heart failure.
3. An 80-year-old woman with a history of diabetes and hypertension with a diagnosis of inferior MI who died after hospitalization in ICU for 72 days because of heart failure.

## 4. Discussion

Staged HCR treatment is a novel and suitable treatment method. Our study was performed between three and seven days (mean of 4.6 days) between PCI and CABG. According to our findings, this is the first study performed between three and seven days in Iran. Thus, this study was conducted to determine the complications of staged HCR in acute STEMI patients. This study was performed on 33 patients diagnosed with acute MI who underwent staged HCR. The mean age of patients was 64.88 ± 9.24 years, with men constituting 23 patients (69.7%). The mean duration of hospitalization was 8.9 ± 11.6 days (3–72 days). The mean ejection fraction and syntax score were 36.5 ± 10.2 and 31.21 ± 6.7, respectively. The major involved vessel of patients was 3VD. The most common target vessel for angioplasty was RCA. The most common type of MI was inferior MI. The most common risk factors in patients were diabetes (36.4%), hypertension (51.5%), dyslipidemia (21.2%), heart failure (6.1%), and CVA (3.1%); also, 87.9% of patients needed to receive pack cells following hybrid surgery. Based on the results of our study, 87.9% of the patients were required to receive packed red blood cells following hybrid surgery, and the mean reception of pack cells in patients was 640.9 ± 670.9 cc. In the study by Ahmet Kagan et al. comparing the two groups of CABG following PCI and the second group of CABG without intervention, consumption of blood products was significantly higher in group I than in group II (3.3 ± 1.8 vs. 1.7 ± 0.9, *p* < 0.001) [16]. Also, the values of blood discharge postoperation were substantially higher in group I than in group II (172 ± 546 vs. 424 ± 185 cc, *p* < 0.001) [16], which is in line with our study. In the present study, none of the study patients had revascularization. In the study performed by Modarau et al., in 2015 on 100 patients undergoing staged HCR, PCI was repeated for three patients [17]. In the study by Halkos et al. in 2013, for 13 out of 300 patients, a second-time cardiovascular intervention was required [14]. In the study by Adams et al. in 2014, 96 patients (13%) needed coronary artery intervention [18]. Also, in the survey by Tajstra et al. in 2018, out of the total 200 patients, 37.2% needed revascularization in the five-year follow-up [19]. In the study by Gasior et al., 200 patients were investigated; over 12 months, the extent of vascular interventions was 2%. None of the five studies concurs with the present research. This can be due to the small sample size [20].

In the present study, none of the patients had CVA after the hybrid surgery in the follow-up, with a mean duration of three years and two months. In the study by Gasior et al. in 2014, no case of CVA was observed in the 200 patients examined in reference [20], in line with our research. Nevertheless, in the study by Modarau et al., one case of CVA out of 100 [17], and in the study by Halkos et al. in 2013, three cases (1%) of 300 cases [14]. In our present study, none of the patients experienced a CVA in the follow-up period, which had a mean duration of three years and two months. This aligns with Gasior et al.'s 2014 study, where no CVA cases were reported among the 200 examined patients [20]. However, Modarau et al. noted one CVA case out of 100 [17], and Halkos et al. observed three cases (1%) out of 300 in 2013 [14]. Tajsra et al.'s 2018 survey found a CVA incidence of 2.1% among 200 patients in the five-year follow-up [19]. None of the three studies accorded with our research, possibly due to the variable follow-up times and sample sizes.

In our study, none of the patients died early (earlier than one month). In the study by Halkos et al. in 2013 [14], out of 300 patients, 4 (1.3%) had early mortality (during the first 30 days), which is different from our research, which can be due to the small sample size.

In our study, in the follow-up with a mean duration of three years and two months, 9.1% of patients had late mortality. In the study by Tajstra et al. in 2018, the mortality in the five-year follow-up out of 200 patients was 6.4% [19], which aligns with our study. In the study by Modarau et al. in 2015 and the study by Gasior et al. in 2014, in one year, one patient out of 100 cases died because of heart failure [17], and out of 200 patients over 12 months, the mortality rate was 2% [20]. None of these two studies concurs with our research, possibly due to the small sample size and the duration of follow-up.

## 5. Conclusion

We obtained acceptable results in this study, which was performed in Iran for the first time. Thus, staged HCR may be considered in indicated patients.

### 5.1. Suggestions

Since it is a new technique and study and has yet to be performed so widely worldwide, it is suggested to conduct further studies with a larger sample size and then compare this group of patients with those who undergo open-heart surgery electively. Also, a comparison should be made on different types of staged HCR regarding the intervals between PCI and CABG (concurrent, 1–7 days apart, and more than seven days apart).

## Figures and Tables

**Table 1:** Average age, BMI, discharge fraction, number of hospitalization days, and syntax score in the studied patients.

Parameter	Mean ± standard deviation
Age (years)	64.88 ± 9.24
Body mass index (kg/m^2^)	24.99 ± 4.38
Discharge ejection fraction (%)	36.5 ± 10.2
Number of hospitalization days	11.6 ± 8.9
Syntax score	31.21 ± 6.7

**Table 2:** The frequency distribution of gender, diabetes, hypertension, dyslipidemia, heart failure, and CVA.

Parameter		Frequency	Percentage (%)
Gender	Male	23	69.7
	Female	10	30.3

Diabetes mellitus	Yes	12	36.4
	No	21	63.6

Hypertension	Yes	17	51.5
	No	16	48.5

Dyslipidemia	Yes	7	21.2
	No	26	78.8

HF	Yes	2	6.1
	No	31	93.9

CVA	Yes	1	3.1
	No	32	96.9

Family history of CAD	Yes	3	9.09
	No	30	90.09

History of previous CAD	Yes	1	3.1
	No	32	96.9

**Table 3:** Number of involved vessels, type of MI, target vessel, syntax score, and the type of implanted stent in the studied patients.

Number of involved vessels	SVD	1	3.1%
	2VD	5	15.1%
	3VD	27	81.8%

Target vessel for angioplasty	LCX	7	21.2%
	LAD	10	30.3%
	RCA	15	45.4%
	PLV	1	3.1%

MI type	Inferior	18	54.5%
	Anterior	12	36.3%
	Posterolateral	1	3.06%
	Anterolateral	1	3.06%
	Inferoposterolateral	1	3.06%

Syntax score	<22	2	6.1%
	33< و >22	22	66.6%
	33<	9	27.3%

Type of implanted stent	Coronary drug-eluting stent	4	12.1%

**Table 4:** Distribution of the frequency of receiving cell packs in the studied patients.

Name of the variable	Status	Frequency	Percentage (%)
Received cell pack	Positive	29	87.9
	Negative	4	12.1

**Table 5:** The average levels of creatinine in the studied patients.

Name of the variable	Mean ± standard deviation (mg/dl)
The mean preoperative creatinine	1.109 ± 0.55
The mean postoperative creatinine	1.164 ± 0.47
The mean changes in creatinine elevation	0.055 ± 0.07

**Table 6:** Frequency distribution of reintervention, arrhythmia, and surgical site infection in studied patients in the follow-up.

Name of the variable	Status		Frequency	Percentage (%)
Reintervention	Positive		0	0
	Negative		33	100

Arrhythmia	Yes		18	5.54
	No		15	5.45

Type of arrhythmia	AF	Yes	6	18.1
		No	27	81.9
	VF	Yes	0	0
		No	33	100
	VT	Yes	1	3.1
		No	32	96.9
	PVC	Yes	11	33.3
		No	22	66.7

Surgical site infection	Yes		2	6.1
	No		31	93.9

**Table 7:** Frequency distribution of CVA, sepsis, need for dialysis, and urinary tract infection in the studied patients.

Name of the variable	Status	Frequency	Percentage (%)
CVA	Yes	0	0
	No	32	100

Sepsis	Yes	0	0
	No	33	100

Need for dialysis	Yes	1	3.1
	No	32	96.9

UTI	Yes	2	6.1
	No	31	93.9

**Table 8:** Frequency distribution of early and late mortality in the studied patients.

Name of variable	Status	Frequency	Percentage (%)
Early mortality	Yes	0	0
	No	33	100

Late mortality	Yes	3	9.1
	No	30	90.9

## Data Availability

The data used to support the findings of this study are available from the corresponding author upon request.

## References

[1] Go A. S., Mozaffarian D., Roger V. L. (2014). American heart association statistics committee and stroke statistics subcommittee, executive summary: heart disease and stroke statistics--2014 update: a report from the American heart association. *Circulation*.

[2] Kelly T., Yang W., Chen C.-S., Reynolds K., He J. (2008). Global burden of obesity in 2005 and projections to 2030. *International Journal of Obesity*.

[3] Kadkhodaei Khalafi M., Dabidi Roshan V., Beyranvand M. R. (2011). Response of the cardiovascular physiological and functional markers following the short term taurine supplementation and burce protocol in patients with cardiac heart failure. *Razi Journal of Medical Sciences*.

[4] Rashidlamir A. (2012). Investigation of the effect of aerobic and resistance exercises on peripheral blood mononuclear cells ABCG1 gene expression in female athletes. *Shahid Sadoughi University_Journals*.

[5] Gelissen I. C., Harris M., Rye K. A. (2006). ABCA1 and ABCG1 synergize to mediate cholesterol export to apoA-I. *Arteriosclerosis, Thrombosis, and Vascular Biology*.

[6] Shokrallahnia-Roshan A., Sadeghi H., Shirani S., Nejatian M. (2013). Effects of strength training and cardiac rehabilitation programs on the biomechanical parameters of blood flow velocity and blood flow rate and its relation with arterial stiffness index in brachial and femoral arteries with Coronary Artery Bypass Grafting Patients (CABG). *Archives of Rehabilitation*.

[7] Hatmi Z., Tahvildari S., Gafarzadeh Motlag A., Sabouri Kashani A. (2007). Prevalence of coronary artery disease risk factors in Iran: a population based survey. *BMC Cardiovascular Disorders*.

[8] Mohammadzadeh N., Safdari R. (2017). Chronic heart failure (CHF) management through agent technology. *Payavard Salamat*.

[9] Kazemi T., Sharifzadeh G. R., Zarban A., Fesharakinia A., Rezvani M. R., Moezy S. A. (2011). Risk factors for premature myocardial infarction: a matched case-control study. *Journal of Research in Health Sciences*.

[10] Weiss A. J., Elixhauser A. (2014). Trends in operating room procedures in US hospitals. https://hcup-us.ahrq.gov/reports/statbriefs/sb171-Operating-Room-Procedure-Trends.pdf.

[11] Paryad E., Rouhi Balasi L. (2018). Smoking cessation: adherence based on patients’ illness perception after coronary artery bypass grafting surgery. *Indian Heart Journal*.

[12] Mirbagheri M., Taghipour H. R., Farhadi N., Mirbagheri L., Imani Foladi A. A., Nourani M. R. (2012). Rapid diagnosis of acute kidney injury (AKI) associated with cardiac surgery, using the liver type fatty acid binding protein (L-FABP) biomarker. *Mljgoums*.

[13] Benedetto U., Raja S. G., Soliman R. F. (2014). Harefield Cardiac Outcomes Research Group. Minimally invasive direct coronary artery bypass improves late survival compared with drug-eluting stents in isolated proximal left anterior descending artery disease: a 10-year follow-up, single-center, propensity score analysis. *Journal of Thoracic Cardiovascular Surgery*.

[14] Halkos M. E., Walker P. F., Vassiliades T. A. (2014). Clinical and angiographic results after hybrid coronary revascularization. *The Annals of Thoracic Surgery*.

[15] Sardar P., Kundu A., Bischoff M. (2018). Hybrid coronary revascularization versus coronary artery bypass grafting in patients with multivessel coronary artery disease: a metaanalysis. *Catheterization and Cardiovascular Interventions*.

[16] As A. K., Engi̇n M., Turk T. (2021). Early-term results of early coronary artery bypass graft surgery in patients undergoing primary percutaneous coronary intervention due to acute coronary syndrome. *The European Research Journal*.

[17] Modrau I. S., Holm N. R., Mæng M. (2015). One-year clinical and angiographic results of hybrid coronary revascularization. *The Journal of Thoracic and Cardiovascular Surgery*.

[18] Adams C., Burns D. J., Chu M. W. (2014). Single-stage hybrid coronary revascularization with long-term follow-up. *European Journal of Cardio-Thoracic Surgery*.

[19] Tajstra M., Hrapkowicz T., Hawranek M. (2018). Hybrid coronary revascularization in selected patients with multivessel disease: 5-year clinical outcomes of the prospective randomized pilot study. *Journal of the American College of Cardiology: Cardiovascular Interventions*.

[20] Gąsior M., Zembala M. O., Tajstra M. (2014). Hybrid revascularization for Multivessel Coronary artery disease. *Journal of the American College of Cardiology: Cardiovascular Interventions*.

